# Ethical considerations of e-cigarette use for tobacco harm reduction

**DOI:** 10.1186/s12931-016-0370-3

**Published:** 2016-05-17

**Authors:** Caroline Franck, Kristian B. Filion, Jonathan Kimmelman, Roland Grad, Mark J. Eisenberg

**Affiliations:** Centre for Clinical Epidemiology, Lady Davis Institute, Jewish General Hospital, McGill University, Montreal, QC Canada; Department of Epidemiology, Biostatistics and Occupational Health, McGill University, Montreal, QC Canada; Division of Clinical Epidemiology, McGill University, Montreal, QC Canada; Biomedical Ethics Unit, McGill University Montreal, Montreal, QC Canada; Department of Social Studies and Medicine, McGill University, Montreal, QC Canada; Department of Family Medicine, McGill University, Montreal, QC Canada; Division of Cardiology, Jewish General Hospital, McGill University, Montreal, QC Canada; Divisions of Cardiology and Clinical Epidemiology, Jewish General Hospital/McGill University, 3755 Côte Ste-Catherine Road, Suite H-421.1, Montreal, Quebec H3T 1E2 Canada

**Keywords:** E-cigarettes, Harm reduction, Ethics

## Abstract

Due to their similarity to tobacco cigarettes, electronic cigarettes (e-cigarettes) could play an important role in tobacco harm reduction. However, the public health community remains divided concerning the appropriateness of endorsing a device whose safety and efficacy for smoking cessation remain unclear. We identified the major ethical considerations surrounding the use of e-cigarettes for tobacco harm reduction, including product safety, efficacy for smoking cessation and reduction, use among non-smokers, use among youth, marketing and advertisement, use in public places, renormalization of a smoking culture, and market ownership. Overall, the safety profile of e-cigarettes is unlikely to warrant serious public health concerns, particularly given the known adverse health effects associated with tobacco cigarettes. As a result, it is unlikely that the population-level harms resulting from e-cigarette uptake among non-smokers would overshadow the public health gains obtained from tobacco harm reduction among current smokers. While the existence of a gateway effect for youth remains uncertain, e-cigarette use in this population should be discouraged. Similarly, marketing and advertisement should remain aligned with the degree of known product risk and should be targeted to current smokers. Overall, the available evidence supports the cautionary implementation of harm reduction interventions aimed at promoting e-cigarettes as attractive and competitive alternatives to cigarette smoking, while taking measures to protect vulnerable groups and individuals.

## Background

Electronic cigarettes (e-cigarettes) have polarized the public health community unlike any previous alternative to smoking. Although their efficacy as smoking cessation aids remains unclear [1], anecdotal evidence suggests that many people have successfully quit smoking with the use of e-cigarettes. Due to their similarity in form and function to tobacco cigarettes, e-cigarettes could play an important role in tobacco harm reduction. However, intense divisiveness has resulted from the absence of conclusive evidence demonstrating product safety for individual and public health. Several ethical issues have been identified pertaining to their use both as recreational products and harm reduction devices, including their potential appeal to non-smokers, their potential to act as a gateway to cigarette smoking, and their potential to renormalize a public smoking culture. To this end, we examined the ethical issues surrounding the availability and use of e-cigarettes for tobacco harm reduction, with the objective of understanding their potential contributions to public health. Specifically, our framework draws upon tensions between utilitarianism and liberalism in public health ethics [2], the former aiming to produce the largest public health gains through the greatest reduction in the burden of disease, and the latter holding paramount individuals' right to self-determination in health.

### The burden of smoking-attributable disease

Cigarette smoking remains the leading cause of preventable mortality worldwide, contributing to the death of approximately 480,000 Americans annually [3]. Smoking also produces substantial morbidity costs: estimates show that 6.9 million Americans reported major smoking-related morbidity in 2009, constituting 10.9 million lifetime cases of smoking-attributable disease [4]. Cessation efforts have largely failed to address the wealth of behavioral and social components to cigarette addiction. The majority of the lifestyle benefits conferred by cigarette smoking, including alertness, focus, stress reduction, and social opportunities [5, 6], are not comparably paralleled with existing smoking cessation therapies. In addition, among the strongest habit-forming properties of tobacco cigarettes are the behavioral cues associated with their use, including regular hand-to-mouth action and the production of smoke [7, 8]. Consequently, there is an urgent need for novel cessation therapies that target both the physiological and behavioral components of cigarette smoking. A device that retains the feel and function of cigarettes and reduces their associated health costs could lead to substantial public health benefits. Given their striking similarity to tobacco cigarettes and their high degree of acceptability among smokers [9–11], e-cigarettes constitute the closest approximation to such a harm reduction device to date.

### The role of tobacco harm reduction in public health

Harm reduction policies attempt to diminish the damaging effects of a particular behavior without aiming to eliminate the behavior itself. Common applications include the provision of needle exchanges and safe injection kits to injection drug users, and the use of methadone to treat opiate addiction. Despite continued resistance to harm reduction interventions, there is strong evidence demonstrating their successes in public health, most notably in reducing the incidence of HIV and Hepatitis C infection [12–14]. Critics may argue that tobacco harm reduction, as it applies to e-cigarettes, remains distinct from harm reduction for other forms of drug addiction. While there is no definitive evidence that either e-cigarettes or needle exchanges promote substance initiation among non-users, critics have expressed concerns about the possibility of a gateway effect of e-cigarettes towards conventional cigarettes [15]. In addition, unlike e-cigarettes, needle exchanges are not backed by powerful political lobbyists or for-profit companies [15]. Lastly, injection drug use is comparably invisible relative to the conspicuousness of using an e-cigarette in public [15]. While these important distinctions highlight the need for closer examination, they do not inherently exclude the harm reduction potential of e-cigarettes.

The burden of smoking-related illness suggests that novel public health interventions designed to reduce the harms associated with cigarette smoking are needed. Virtually all interventions to date have focused on eliminating nicotine use, as standard nicotine replacement therapies are indicated for use up to 12 weeks [16]. These successes have been limited, with just over 15 % of smokers motivated to quit achieving prolonged abstinence at 12 months with the aid of a smoking cessation therapy [17]. Despite the fact that an elimination-centered approach is incongruous with the understanding that harm reduction strategies are more practical and feasible than enforcing population-wide abstinence [18], anti-tobacco activists have expressed concern that harm reduction might overshadow cessation messages, effectively resulting in a reduction in the number of successful quitters [19].

Tobacco harm reduction continues to be met with skepticism by public health advocates [20] whose distrust of safer smoking products dates back to a misguided endorsement of “light” cigarettes in the 1950’s and 60’s [18, 21]. More recently, critics denounced the use of low-nitrosamine smokeless tobacco products, commonly known as “snus,” for tobacco harm reduction despite evidence that the increased use of snus among Swedish men was accompanied by a reduction in the prevalence of cigarette smoking and tobacco-related disease [22, 23]. Arguments against the use of smokeless tobacco for harm reduction are similarly used against e-cigarettes, including the continued promotion of an addictive substance, uncertain long-term safety concerns, the possibility of a gateway effect to conventional tobacco products, and concerns about questionable terms of engagement with the tobacco industry [24]. An important distinction between e-cigarettes and smokeless tobacco to be considered among public health critics is the former’s inherent likeness to conventional cigarettes, which arguably increases their appeal as an alternative to knowingly harmful combustible products. However, this distinction has not prevented significant controversy and debate in the United Kingdom, stemming from polarized opinions concerning the strength of the evidence regarding e-cigarettes’ potential for harm [25].

The principal quandaries in framing e-cigarettes as a tool for harm reduction occur first in determining whether it is morally objectionable to promote a product whose long-term health effects remain unknown; second, in establishing whether mitigating a harm that already exists is morally superior to preventing a same or similar harm from materializing [26]. What is the government’s role in regulating and potentially incentivizing these products? Should physicians encourage tobacco harm reduction by advocating for the use of e-cigarettes? As they are neither tobacco products nor approved cessation devices, e-cigarettes constitute a novel product whose harm reduction potential stands to be weighed against the ethical implications surrounding their availability and use.

## Review

### E-cigarette safety

E-cigarettes typically contain a solution of propylene glycol or glycerin, with or without nicotine, that is vaporized upon inhalation by the user [27]. Unlike tobacco cigarettes, e-cigarettes are free of combustion [28], the mechanism through which toxicants contained in burned tobacco are inhaled and absorbed by the user [3]. To date, biochemical studies of e-cigarettes have failed to raise any serious health concerns [3, 20]. The most frequently reported adverse events associated with their use have included nausea, throat and mouth irritation, headache, and dry cough, all of which were found to resolve over time [3, 29]. Although e-cigarettes are believed to have similar toxicity as existing nicotine replacement therapies [20], the generalizability of these findings remains unclear given the absence of standardized manufacturing practices and the proprietary nature of industry studies. The product’s novelty also entails that there is insufficient data to judge the long-term effects of regular inhalation of propylene glycol or glycerin. However, studies of artificial smoke generators concluded that exposure to propylene glycol mist can cause ocular and upper airway irritation [30], which could potentially be of concern among users with chronic lung disease, including asthma, emphysema, or bronchitis [31].

Safety evaluations will require quantifying the degree of risk warranted in the face of incomplete evidence with which to inform decision-making. In turn, promoting autonomy, or the right to make individual decisions with regards to one’s life choices, requires the provision of information concerning the risks and benefits associated with a given behaviour and with voluntary choice [32]. This rights-based position is compelling given that the majority of e-cigarette users are current smokers attempting to quit or reduce their number of cigarettes smoked [33]. While autonomy may be compromised through the influence of nicotine addiction, the consequences may be less pronounced where this choice consists of selecting between alternative sources of nicotine (of potential equal or similar satisfaction), rather than choosing between indulgence and abstinence. However, were the demographics of e-cigarette users to change, for instance through an increased number of non-smokers or youth taking up e-cigarettes, from a utilitarian perspective, the autonomy argument may become less convincing in weighing individual harm against public good.

### Efficacy for smoking cessation and reduction

The best evidence concerning the efficacy of e-cigarettes for smoking cessation and reduction is presented in a 2014 Cochrane review [34] that examined 13 studies, two of which were randomized controlled trials (RCTs) [11, 35]. While the included studies found some evidence that e-cigarettes help smokers quit or reduce smoking, the authors concluded that a lack of high-quality RCTs reduces the certainty of these effects. Nonetheless, available data from several observational studies suggest that e-cigarettes can lead to substantial smoking reduction among smokers not motivated to quit [36–38]. Many smokers continue to engage in dual use of e-cigarettes and tobacco cigarettes. A study examining the effects of cigarette reduction on cardiovascular risk factor levels in regular smokers (15–45 cigarettes per day) motivated to decrease their consumption demonstrated that reducing the number of self-reported cigarettes per day by at least 40 % led to significant improvements (*p* < 0.05) in several biomarkers of cardiovascular disease [39]. However, these were only modestly correlated with a reduced risk of disease. Similarly modest risk reductions found in other studies have led researchers to hypothesize that cigarette reduction among heavy smokers is frequently accompanied by compensatory smoking behavior, including prolonging the duration of each cigarette smoked [40–42]. Thus, despite improvements in biomarkers including hemoglobin, leukocyte counts, fibrinogen, and cholesterol, there is no evidence that reducing smoking to as few as ten cigarettes per day produces improvements in clinical cardiovascular disease outcomes [3].

The absence of improved cardiovascular outcomes, however, does not preclude the existence of benefits attributed to reduced smoking. A population-based cohort study with up to 31 years of follow-up determined that reducing smoking from 20 to fewer than ten cigarettes per day produced a 27 % (95 % confidence interval [CI], 2–46 %) reduction in the relative risk of lung cancer as compared to continuously smoking more than 15 cigarettes per day [42]. In a second study, smokers unwilling to quit were randomized to either 4 weeks of reduced smoking with subsequent advice to quit or to usual care with only quit advice [43]. Both groups had similar quit rates at 6 months, suggesting that reduction messages do not hinder cessation attempts. Similarly, a review of 19 controlled, cohort, case–control, and experimental studies examining the impact of reduction messages on smoking cessation revealed no study concluded that smoking reduction decreases subsequent smoking cessation among smokers unwilling to quit [44]. Rather, reduced smoking likely constitutes a first step to attempt and subsequently achieve abstinence, particularly among smokers who perceive themselves as unable to quit [39].

### Use of e-cigarettes among non-smokers

A key challenge faced by regulatory agencies in choosing how to regulate e-cigarettes rests in considering the possibility of increased use among non-smokers. Data from a 2010–2013 online survey of US adults conducted in samples ranging from 2,505 (in 2010) to 4,170 (in 2012) respondents revealed that ever use of e-cigarettes was highest among current and former cigarette smokers compared to never smokers in every survey year [45]. Specifically, the proportion of never cigarette smokers who reported ever use of e-cigarettes was 1.3 % in 2010, 1.3 % in 2011, 2.3 % in 2012, and 1.2 % in 2013. Similarly in 2012, just 0.2 % of never smokers reported using an e-cigarette in the past 30 days. The increase in e-cigarette awareness (40.9 to 79.7 %) and ever use (3.3 to 8.5 %) among all US adults between 2010 and 2013 thus appears to be driven by current and former smokers. At present, it is unclear what proportion of use among former smokers can be attributed to recent quitters’ attempts to manage their cessation efforts, or to successful quitters newly initiating e-cigarettes. However, due to their frequent use as unapproved smoking cessation aids [10], it is likely that many former smokers are also recent quitters.

Concerns have been raised that higher rates of never smokers initiating e-cigarettes would result in net public health harms via increased nicotine addiction, and the possibility for e-cigarettes to act as a gateway to tobacco cigarettes. There is limited evidence that nicotine exerts a priming effect on brain circuitry, which helps to explain why nicotine is frequently used as a precursor to other hard drugs [46]. However, the implications of such priming are unclear, particularly as concerns a possible gateway effect of e-cigarettes to tobacco cigarettes. Tenets of economics dictate that risk-minimizing strategies, including sunscreen, condoms, and travel vaccines, encourage more people to engage in otherwise risky activities [6]. The same should be expected of e-cigarettes, probably leading to eventual high product uptake among non-smokers.

**Fig. 1:**
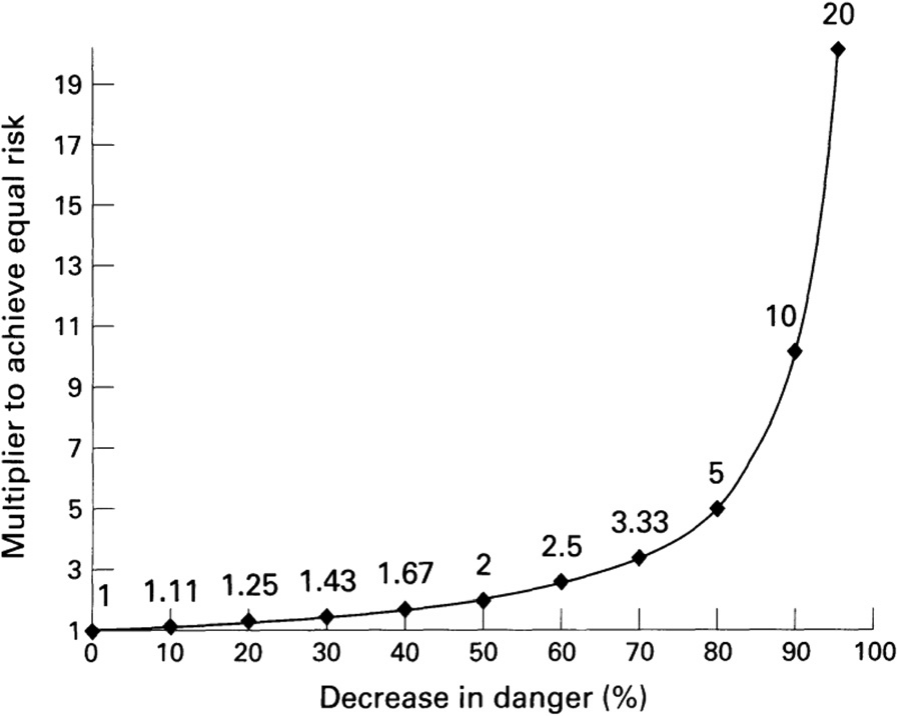
The risk/use equilibrium. Each point on this curve indicates the multiplier needed to achieve a constant level of population risk, given specific levels of decreased danger per user. For example, if 100 individuals used a product with full danger (for example, killing 100 % of users), 10 times that number (1000 individuals) would need to use a product that had 90 % decreased danger, to achieve an equal health problem (100 dead in each instance). The formula is *Y* = 100/100-X, where *Y* = multiplier and *X* = decrease in danger, expressed in percentages. If danger is 0.1 %, use would have to increase by 1000 times to produce a problem of the same magnitude as the full risk product (not plotted on figure). For a given risk on the curve, use that is increased by a smaller multiplier represents a public health benefit, and use that is increased by a larger multiplier represents a public health (population level) cost. Figure and legend reproduced from [47] with permission from BMJ Publishing Group Ltd.

A useful paradigm that reconciles liberalism and utilitarianism in illustrating the impact of displacing a high-risk activity with a low-risk one is the *risk/use equilibrium* (Fig. 1) [47]. For instance, if e-cigarettes reduced a smoker’s risk by 99 %, for every smoker who switched to e-cigarettes, 100 non-smokers would need to initiate e-cigarettes to attain no net public health benefit. Were e-cigarettes so little as 95 % less harmful than tobacco cigarettes, 20 % of non-smoker uptake of e-cigarettes would be required to offset the public health benefits of 1 % of smokers switching to e-cigarettes, generally representing the upper limit of nicotine usage prevalence worldwide [6]. Consequently, it is unlikely that e-cigarettes would result in net public health harms despite the inevitable uptake of the product in a non-smoking fraction of the population. This framework provides nuance to the absolutist position that any non-smoker uptake of e-cigarettes would have overall adverse effects on population health. In practice, sound public health policy can sustain autonomous choices with deleterious consequences to the extent that these do not outweigh net public health benefits.

It is important to consider how the risks and benefits of tobacco harm reduction are differentially experienced by disadvantaged populations. It is well documented that cigarette smoking is associated with structural, material, and perceived socioeconomic disadvantage [48, 49]. Although e-cigarettes could increase health disparities if used differentially for harm reduction, given their comparable cost to tobacco cigarettes, for which smokers have already found the income to purchase, they are unlikely to increase disparities in practice. However, this remains an issue that requires continued surveillance to better understand practices in different socioeconomic groups.

### Use of e-cigarettes among youth

Nicotine liquid (e-liquid) flavourings are widely available in youth-friendly flavors, including strawberry, bubble gum, and chocolate. Flavored tobacco has been shown to have a large market share among youth aged 12 to 17 years [50], confirming the attractiveness of these products to new and young smokers and their likely contribution to smoking initiation. The appeal of flavorings is particularly disconcerting given an increase in United States (US) reports of accidental e-cigarette exposure (including exposure to e-liquid) in children [51], with many bottles of e-liquid containing several times the lethal dose of nicotine in children [52]. Previous studies also highlight positive youth perceptions and expectations of flavored tobacco products, namely that they are both better-tasting and safer than non-flavored tobacco products [53]. The combination of added flavor to a device that is also perceived to be less harmful than tobacco cigarettes is likely to entice youth to use e-cigarettes. Given the unknown health effects of long-term nicotine use [3] and inhaled propylene glycol [54], the safety profile of even the most reliable e-cigarette is yet unknown, and the consumption of nicotine among youth remains undesirable. For this reason, there is widespread consensus concerning attempts to restrict e-cigarette sales to youth in the US [55].

The gateway hypothesis has its specific application to youth, for whom the balance of potential benefits and risks associated with harm reduction must also be considered. Although the association between cigarette smoking and e-cigarette use has recently been examined in a cross-sectional study of adolescents [56], given the study design and its temporal ambiguity, it is difficult to draw firm conclusions from these data [57]. As it is unclear whether youth who use e-cigarettes are more likely to use tobacco cigarettes as a consequence of the e-cigarette itself, there remains a need to evaluate the gateway hypothesis in this population over time.

### Marketing and advertising

An extension of youth protection is the question of regulating advertisement and marketing to broad audiences. Comprehensive advertising bans would likely minimize any perceived government endorsement of e-cigarettes. However, the inadvertent message sent to consumers by regulating e-cigarettes as strictly as tobacco cigarettes may be that these products are comparably, if not equally harmful. E-cigarettes are likely to be considerably less toxic than tobacco cigarettes [31] given the absence of tobacco combustion inherent to cigarette smoking, which releases pulmonary carcinogens including polycyclic aromatic hydrocarbons, *N*-Nitrosamines, and various other cytotoxic compounds [58]. From both utilitarian and liberal perspectives, misinformation through the provision of inaccurate comparative risk is fundamentally unethical for its failure to allow consumers to make informed choices, and for effectively conveying the message that smokers may as well continue to smoke [59].

As marketing and advertisement play an important role in the public’s perception of e-cigarettes, governments have an ethical duty to ensure that the product’s media portrayal is appropriately aligned with its known degree of risk. If the public health community’s aim is to market e-cigarettes to current smokers, it follows that advertisements should have at least equal reach to this target audience as tobacco cigarettes. This strategy, termed “levelling up,” would allow e-cigarettes to be sold and marketed similarly to conventional tobacco products, as well as benefiting from the possibility of lower tax rates owed to their reduced potential for harm [60]. However, the relative absence of restrictions to date in the US has led e-cigarette marketing to permeate most media outlets through the likes of celebrity endorsements, images associated with youth culture, and statements encouraging consumers to reclaim lost freedoms [61]. Importantly, today’s youth have never known mass marketing of a recreational nicotine product [31]. In 2014, the World Health Organization released a statement encouraging government bodies to restrict e-cigarette promotion and sponsorship, including ensuring that any advertisement does not target youth, non-smokers, or people not using nicotine [62]. However, because e-cigarettes are not currently regulated as tobacco products in the US, they are neither subject to clear nor comprehensive regulations.

### Use of e-cigarettes in public places

Ethical concerns surrounding second-hand vaping stem from the unknown health effects of vaporized e-liquid in the presence of potentially vulnerable bystanders. Although e-cigarettes emit significantly fewer toxins than tobacco cigarettes [63], vaporized e-liquid produces ultrafine particles and volatile organic compounds, including nicotine, which are released into the surrounding air [64]. One study concluded that aerosolized ingredients contained in e-liquid should be of little concern to bystanders as their exposure is likely orders of magnitude lower than that of e-cigarette users and is unlikely to produce adverse health effects [54]. However, studies examining the cytotoxicity of e-liquid flavorings found toxicity to be greater in undifferentiated embryonic stem cells relative to human pulmonary fibroblasts [65], raising potential concerns about exposure risks for pregnant women [31]. Beyond any immediate emission concerns however, the ethical arguments surrounding second-hand vapor exposure are those that apply to tobacco cigarettes: exposure to e-cigarettes should not be imposed upon those who do not choose to use them, providing a strong argument for use restrictions in public places.

### Renormalization of a smoking culture

E-cigarettes theoretically have the potential to subvert decades’ worth of anti-smoking efforts by renormalizing the act of public smoking and the visual presence of smoke-like vapor. This phenomenon could unintentionally encourage the acceptability and eventual uptake of tobacco cigarettes. However, the likelihood of such a phenomenon is difficult to assess and is premised upon e-cigarettes’ potential to act as a gateway to cigarette smoking. One possibility is that the increased conspicuousness of smoke-like vapor may sustain cigarette smoking among smokers who might otherwise have quit [66]. Conversely, the growing acceptability of e-cigarettes could increase pressure on current smokers to quit tobacco cigarettes by virtue of these becoming perceived as socially undesirable predecessors of a “cleaner,” smoke-free device. This question should be continuously revisited as the long-term implications of e-cigarette use become increasingly clear.

### Market ownership

As cigarette companies have acquired the largest e-cigarette brands, they currently benefit from a dual market of smokers and e-cigarette users while simultaneously presenting themselves as agents of harm reduction [31, 67]. This raises concerns about the appropriateness of endorsing a product that directly profits the tobacco industry. Importantly, profit alone is unlikely to increase their market share, particularly in the highly restrictive regulatory environment in which tobacco companies operate. In addition, the unequivocal refusal to associate with the tobacco industry which appears, if only for self-serving reasons, to support tobacco harm reduction [68], could unintentionally damage the credibility of the tobacco control community. Regardless of their industry ownership, e-cigarette companies would nevertheless have a vested interest in maximizing the number of long-term product users. The ethical onus then falls on governments to restrict the influence of industry through appropriate regulations targeting product manufacturing, availability, and use, devised in light of public health interests.

### Directions for future research

There is an urgent need for data from high-quality RCTs to establish the efficacy and safety of e-cigarettes for smoking cessation and harm reduction. In addition, longitudinal studies are needed to monitor product awareness and use among various demographics and to further inform discussions concerning the potential of e-cigarettes as tools for tobacco harm reduction. We identified the primary research questions relevant to the ethical considerations of e-cigarette use for tobacco harm reduction (Table 1), the answers to which would clarify the major ambiguities concerning their optimal regulatory framework. Until such study data become available, governments have an ethical responsibility to enforce regulations to discourage product use among youth and to ensure that product restrictions are devised with public health goals in mind. Available evidence therefore supports the cautionary implementation of harm reduction interventions aimed at promoting e-cigarettes as attractive and competitive alternatives to cigarette smoking, while taking measures to protect vulnerable groups and individuals.

**Table 1:** Ethical considerations surrounding the availability and use of e-cigarettes

Ethical considerations	Supporting arguments	Opposing arguments	Questions to direct future research
Tobacco harm reduction			
Potential for smoking cessation	E-cigarettes may be as effective as the nicotine patch.	Inconclusive evidence of efficacy for smoking cessation.	What is the efficacy of nicotine and non-nicotine e-cigarettes for smoking cessation and reduction?
Potential for smoking reduction	Demonstrated in multiple studies.	Unlikely that cigarette reduction results in significant health benefits.	What is the long-term impact of dual use of e-cigarettes and tobacco cigarettes on health outcomes?
Product safety			
Potential for long-term adverse effects	Unknown impact of long-term propylene glycol inhalation.	No documented serious adverse events to date.	What are the long-term safety implications of nicotine and non-nicotine e-cigarette use?
	Propylene glycol inhalation causes short-term respiratory irritation.		
Autonomy to use a product of unknown risk	Ethical imperative given informed consent.	Public health concerns trump individual rights.	How should consumer rights be weighed against public health concerns?
Use among non-smokers			
Potential to lead to nicotine addiction	Perceived harmlessness may lead never smokers to initiate e-cigarettes.	No evidence for increased nicotine addiction to cause net public health harms.	What is the long-term health impact of nicotine addiction?
Potential gateway effect	Nicotine acts as a priming agent for the brain.	Unclear implications for transitioning to tobacco cigarettes.	How many non-smokers initiating e-cigarettes transition to other tobacco products, including cigarettes?
Use among youth			
Potential to lead to nicotine addiction	Minors require protection.	No evidence of increased nicotine addiction causing net public health harms.	How many youth initiating e-cigarettes report continuous long-term product use (1 year or longer)?
	E-liquid flavorings are attractive to youth.		
Potential gateway effect	Nicotine is a priming agent for the brain.	Unclear implications for transitioning to tobacco cigarettes.	How many youth initiating e-cigarettes transition to other tobacco products, including cigarettes?
Nicotine poisoning among children	Increased calls to poison control centers.	None.	To what extent can the risk of nicotine poisoning among children be mitigated?
	E-liquid flavors are appealing to youth.		
Use in public places			
Potential for passive vaping	Stem cell cytotoxicity.	Limited evidence that passive vaping poses significant health concerns.	What is the long-term impact of passive vaping and second-hand vapor exposure?
	Aerosolized nicotine emissions.		
Renormalized smoking culture			
Potential to subvert decades of anti-smoking efforts	Increased acceptability of smoke-like vapor and smoking behavior.	No evidence that e-cigarettes would be conflated with tobacco cigarettes.	How are the increased awareness and use of e-cigarettes affecting perceptions of cigarette smoking?
Market ownership			
Unethical collaboration with the tobacco industry	Public health endorsement of e-cigarettes increases tobacco company market share.	Possible necessity to collaborate with the tobacco industry to achieve public health gains.	What are the public health implications of tobacco industry ownership of major e-cigarette brands?

## Conclusions

In light of incomplete information concerning the safety and efficacy of e-cigarettes as smoking cessation aids, thresholds of reasonable risk must be established through a frequently revisited balance of probable benefits and harms with which they are associated. Their exponential growth in consumer markets has outpaced the development of an ethical framework with which to establish the appropriate conditions for their availability and use. Current evidence suggests that e-cigarettes have the potential to make significant public health gains through their role as tobacco harm reduction devices. In clinical practice, physicians have an ethical duty to provide their patients with evidence-based comparative risk assessments to allow them to make informed choices with respect to their smoking status. At its core, the objective of the smoking cessation agenda should be to improve population health, which will likely require some concessions in the form of harm reduction. This entails a willingness to negotiate the tensions between utilitarian and liberal ethics in designing policy that upholds autonomy while protecting broader public health interests. Although caution in this regard is requisite, caution alone should not obstruct the ethical imperative to explore the product’s potential further.
